# The effects of melatonin versus placebo on delirium in hip fracture patients: study protocol of a randomised, placebo-controlled, double blind trial

**DOI:** 10.1186/1471-2318-11-34

**Published:** 2011-07-05

**Authors:** Annemarieke de Jonghe, Barbara C van Munster, Hannah E van Oosten, J Carel Goslings, Peter Kloen, Carolien van Rees, Reinder Wolvius, Romuald van Velde, Marcel M Levi, Joke C Korevaar, Sophia E de Rooij

**Affiliations:** 1 Academic Medical Centre, University of Amsterdam, Department of Internal Medicine, Geriatrics section F4-218, Meibergdreef 9, 1105 AZ, Amsterdam, The Netherlands; 2 Gelre ziekenhuizen, Department of Geriatrics, Albert Schweitzerlaan 31, 7334 DZ Apeldoorn, The Netherlands; 3 Academic Medical Center, University of Amsterdam, Trauma Unit Department of Surgery G4-111, Meibergdreef 9, 1105 AZ, Amsterdam, The Netherlands; 4 Academic Medical Center, University of Amsterdam, Department of Orthopaedic Surgery G4-222, Meibergdreef 9, 1105 AZ, Amsterdam, The Netherlands; 5 Tergooihospitals, Department of Geriatrics, Van Riebeeckweg 212, 1213 XZ Hilversum, The Netherlands; 6 Tergooihospitals, Department of Orthopaedic Surgery, Van Riebeeckweg 212, 1213 XZ Hilversum, The Netherlands; 7 Tergooihospitals, Department of surgery, Van Riebeeckweg 212, 1213 XZ Hilversum, The Netherlands; 8 Department of Internal Medicine; Academic Medical Centre, University of Amsterdam, Amsterdam, The Netherlands; 9 Stichting NIVEL, Otterstraat 118-124, 3513 CR Utrecht, The Netherlands

## Abstract

**Background:**

With an ageing population, older persons become a larger part of the hospital population. The incidence of delirium is high in this group, and experiencing delirium has major short- and long-term sequelae, which makes prevention crucial. During delirium, a disruption of the sleep-wake cycle is frequently observed. Melatonin plays an important role in the regulation of the sleep-wake cycle, so this raised the hypothesis that alterations in the metabolism of melatonin might play an important role in the development of delirium. The aim of this article is to describe the design of a randomised, placebo controlled double-blind trial that is currently in progress and that investigates the effects of melatonin versus placebo on delirium in older, postoperative hip fracture patients.

**Methods/Design:**

Acutely hospitalised patients aged 65 years or older admitted for surgical repair of hip fracture are randomised (n = 452) into a treatment or placebo group. Prophylactic treatment consists of orally administered melatonin (3 mg) at 21:00 h on five consecutive days. The primary outcome is the occurrence of delirium, to be diagnosed according to the Confusion Assessment Method, within eight days after start of the study medication. Secondary outcomes are delirium severity, measured by the Delirium Rating Scale; duration of delirium; differences in subtypes of delirium; differences in total length of hospital stay; total dose of antipsychotics and/or benzodiazepine use during delirium; and in-hospital complications. In the twelve-month follow up visit, cognitive function is measured by a Mini-Mental state examination and the Informant Questionnaire on Cognitive Decline in the Elderly. Functional status is assessed with the Katz ADL index score (patient and family version) and grip strength measurement. The outcomes of these assessments are compared to the outcomes that were obtained during admission.

**Discussion:**

The proposed study will contribute to our knowledge because studies on the prophylactic treatment of delirium with long term follow up remain scarce. The results may lead to a prophylactic treatment for frail older persons at high risk for delirium that is safe, effective, and easily implementable in daily practice.

**Trial registration:**

Dutch Clinical Trial Registry: NTR1576

## Background

Delirium is the most frequent neuropsychiatric syndrome observed in acutely admitted elderly patients in hospitals and is associated with an increased risk of dementia, mortality, and institutionalisation [1]. The incidence of delirium varies depending on the methods employed and the population studied but were shown to be 53% in a former cohort of hip fracture patients studied by our group [2]. Most clinical patients with delirium suffer from pre-existing cognitive impairment based upon neurodegenerative brain lesions. The prophylactic treatment or early treatment of delirium may prevent these patients from experiencing accelerated cognitive decline [3]. Thus, prevention is crucial.

The current prophylactic treatment of delirium consists of both pharmacological treatment with antipsychotics and non-pharmacological interventions, of which the latter seems the most effective [4]. Pharmacological treatment with antipsychotics often leads to serious side effects such as drowsiness and concomitant falls. In addition, up to a ten-fold increase in risk of cerebrovascular accidents in the first week of treatment was found and a higher frequency of prolonged QT-interval or extra pyramidal symptoms have been reported [5,6]. Given these serious side effects, safer treatment options are warranted. Recently, melatonin has been hypothesised to play a part in delirium, both as a biomarker and as a safe treatment option [7].

Melatonin, a hormone secreted by the pineal gland, plays an important role in the regulation of the sleep-wake cycle [8]. During delirium, disruption of the sleep-wake cycle, such as fragmented sleep during the night and sleepiness during the day, is frequently observed [9]. This trend raises the hypothesis that alterations in the metabolism of melatonin may play an important role in the development of delirium [10]. Elderly persons have decreased peaks of nocturnal melatonin concentrations [11]. Serum melatonin concentrations were also found to be lower with later nocturnal peak concentrations in elderly persons with insomnia as compared to age-matched controls without insomnia [12]. Additionally, a disturbed circadian pattern of melatonin secretion has been found in post-operative patients with delirium [13]. In another study among medical patients, differences in urinary melatonin metabolite concentrations were found during delirium and after recovery from delirium [14]. These results may suggest an association between delirium and a disturbed melatonin secretion pattern.

Investigations into the relationship between melatonin therapy and delirium have also been undertaken. One case report showed that exogenous administration of melatonin prevented the occurrence of delirium [15]. There are indications that melatonin is effective in the treatment of sleep-wake cycle disturbances in ICU patients and in patients with dementia [16,17]. Moreover, it has been demonstrated that treatment with melatonin increases total sleeping time and reduced sleep latency in elderly patients [18], and it enhances sleep time and night activity in patients with Alzheimer's dementia [19]. Recently, two randomised clinical trials on prophylactic treatment with melatonin have been performed. Both studies showed a reduction in the occurrence of delirium in medical and elective surgical patients [20,21]. However, no follow up data were available, and the potential of melatonin to prevent long term adverse events like dementia still remains unclear.

This paper describes the design of the study; "The effects of melatonin versus placebo on delirium in hip fracture patients", a trial currently in progress. The aims of the study are to investigate whether prophylactic treatment with melatonin will lead to a reduction in the occurrence of delirium in hip fracture patients and to study whether melatonin prevents long-term adverse outcomes.

## Methods and design

### Research questions

#### Primary research question

1] Is there a difference in the occurrence of delirium within eight days of hospital admission between hip fracture patients treated with melatonin or placebo?

#### Secondary outcome measures

2] Severity and duration of delirium.

3] Subtypes of delirium.

4] Length of hospital stay.

5] Additional use of benzodiazepines during delirium.

6] Total dose of haloperidol for the treatment of delirium.

7] In-hospital complications.

8] Cognitive and functional decline twelve months after hospital admission.

9] Mortality during hospital stay and twelve months after hospital admission.

### Study design

**Figure 1:**
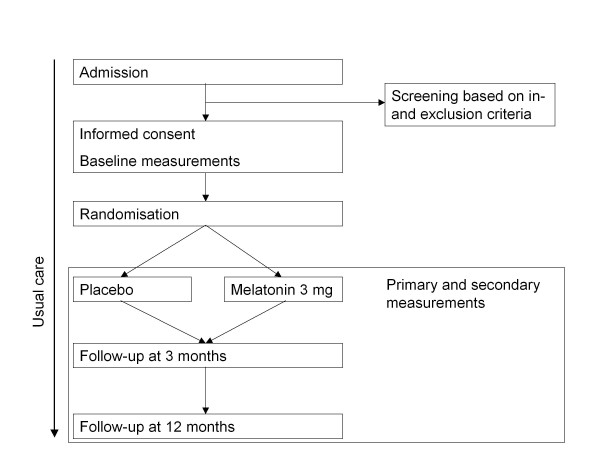
**Design of the RCT; inclusion of patients with hip fracture, 65 years and older**.

This study is a randomised, placebo-controlled, double blind, national, multicentre study with a one-year prospective follow-up. Figure 1 shows the design of the study. Informed consent is obtained from all patients or a legal representative in the case of cognitive impairment. Over five evenings, the patients receive the study medication consisting of a tablet containing either a placebo or 3 mg melatonin. Subsequently, daily assessment for the presence of delirium is performed, and the patients are followed for another three days. In total, each patient is observed for a minimum of eight days. For patients who are not delirious on day eight, daily assessments are stopped. For patients who are delirious by day eight, to evaluate duration of delirium, daily clinical assessments are continued until the symptoms of delirium are resolved or until the patient is discharged. Possible confounding factors including demography, fracture characteristics, type of anaesthesia, type of surgery and peri- and postoperative complications are registered for all patients. This study is carried out in compliance with the Helsinki Declaration. The Medical Ethics Committee (METC) of the Academic Medical Centre (AMC) has approved the study design, protocol and informed consent procedures. The executive board of Tergooihospitals has provided local feasibility approval.

### Location and setting

The primary trial location is the surgical, orthopaedic or trauma surgery ward of the AMC in Amsterdam, The Netherlands. This is a major teaching hospital, containing 1002 beds. The secondary location is Tergooihospitals, a teaching hospital consisting of 630 beds at two locations, in Hilversum and Blaricum, The Netherlands.

### Study population and recruitment

The study population consists of patients of 65 years and older who are admitted for the surgical treatment of hip fractures. Hip fractures are defined as fractures of the femoral neck, the trochanteric region or the proximal femur. Further inclusion criteria are enrolment within 24 hours of admission and the patient must be willing and medically able to receive the study medication according to the protocol for the duration of the study. Exclusion criteria are the inability to speak or understand Dutch, the concomitant use of melatonin and prior participation in this study.

### Randomisation

A randomisation schedule, made by an independent statistician, is employed that is stratified by study centre and uses block randomisation of ten participants per block. The trial-pharmacist is the only one in possession of the randomisation list.

### Study medication

Study medication tablets all look the same and are packaged in small pots labelled according to Good Manufacturing Procedure (GMP) guidelines. The concentration is a fixed concentration as both placebo and melatonin are manufactured by and obtained from the same pharmaceutical company. On prescription by SdR, AdJ or CvR, a pot can be collected at the pharmacy when a patient is included.

### Data collection

Demographic data, medical history and medication use are recorded. Once included in the study, patients are visited daily (and once during the weekend) for efficacy and safety evaluations by an experienced team of geriatric nurses and geriatricians. For the diagnosis of delirium, the Cognitive Assessment Method (CAM) is used [22,23]. The CAM was developed in 1990 and is a validated and widely used screening tool for the presence of delirium. When patients are delirious, the severity of delirium is examined using the Delirium Rating Scale-Revised-1998 (DRS-R-98). The severity of delirium is expressed as the maximum DRS-R-98 score during the delirium period. Subtyping is examined using the Delirium Symptom Interview [24] and the Delirium Motoric Checklist [25]. Cognitive and functional status and the presence of impairment prior to admission are assessed. Each patient and their primary caregiver are asked to complete questionnaires to assess the patient's pre-existent functional and cognitive state two weeks prior to hospital admission. Functional status is assessed using the 15-item Katz Activities of Daily Living (ADL) index score, based on the situation two weeks prior to admission, to be completed by the patient or their closest relative in cases of cognitive impairment. Impairment is calculated using the number of impaired ADLs [26].

Primary caregivers are asked to complete the Informant Questionnaire on Cognitive Decline short form (IQCODE-sf) [27] by recalling the situation two weeks prior to the hip fracture and comparing this with the situation ten years earlier. Patients with a mean score of 3.9 or higher are considered to have global cognitive impairment [28]. Grip strength of the dominant hand, as an objective measurement of functional status [29], is measured using hand-held dynamometry. The severity and number of comorbidities is scored using the Charlson comorbidity index [30]. At the time of discharge from the hospital the total amount of haloperidol and other antipsychotics used during hospital admission is recorded, also the length of stay and in-hospital complications are collected.

### Follow-up assessments

Three months after admission, a home visit is made by a trained nurse, to perform a Mini-Mental State Examination (MMSE) [31] and to measure the grip-strength of the dominant hand. The MMSE is a validated 30-point questionnaire-based test that is used to screen for cognitive impairment. Twelve months after the hospital admission, the patient is visited again at home by a trained nurse to perform a MMSE test and a Katz ADL index score and to assess grip-strength. Additionally, the patient's primary caregiver is asked to complete the caregiver's version of the Katz ADL index score and the IQCODE-sf by telephone.

### Delirium treatment

All patients receive 'care as usual', which means that if postoperative delirium occurs, patients are treated according to standard procedures and assessed for delirium severity and duration. Any underlying illness is treated according to the hospital guidelines. If there is a clinical diagnosis of hyperactive or mixed delirium, treatment with anti-psychotics (most frequently with haloperidol) is started according to a fixed scheme. Daily adjustment of medication takes place depending on the clinical judgement of the consulting geriatric team and/or the attending physician. Antipsychotic medication is standardised as much as possible, with escape medication in cases of acute aggravation. Patients on psychiatric medication, apart from their delirium medication, can continue their prescriptions throughout the study-period because melatonin does not interfere with any other medication.

### Power analysis

The primary endpoint is the occurrence of delirium during the first eight days after the start of study medication. In the AMC, the incidence of delirium in patients after surgical repair of hip fracture is now over 50% [2]. The prevention of delirium has been previously assessed in several studies. At the time this study began, other studies using medication prophylaxis had not shown any significant reduction in the incidence of delirium. Assuming an absolute reduction of 13%, with a 50% incidence of delirium in the control group and an incidence of 37% in the intervention group, we require 226 patients per group (452 patients in total) to detect differences with a power of 80% and a two-sided alpha of 0.05.

### Statistics

Data will be primarily analysed according to the intention-to-treat principle and secondarily compared by per protocol analysis. To study the effectiveness of the melatonin treatment, the difference in the occurrence of delirium between patients treated with or without melatonin will be tested with Chi-square statistics. Continuous secondary outcome measures, if normally distributed, will be tested using a Students' t-test, otherwise Mann-Whitney tests will be employed. The duration of delirium will be tested using a log-rank test. Categorical outcome variables will be tested with a Chi-square test. Outcomes for patients who had delirium at day one of the study will be calculated separately. Subsequently, multivariable logistic regression will be used to compare differences between melatonin and placebo groups in the primary and in the secondary outcome measures.

## Discussion

With an ageing population, older persons become a larger part of the hospital population. Experiencing delirium has major consequences for these patients' outcomes following hospital stay. From treatment trials with haloperidol, it became known that once delirium exists, it is difficult to influence its duration [32]. The prevention of delirium should therefore be a main focus in the treatment of older persons in hospital. To be able to adequately compare the placebo versus melatonin intervention, we selected an RCT-design. Because the proposed study is a prevention trial, the primary measure is 'occurrence of delirium'. We decided to enrol acutely admitted hip fracture patients because they represent a homogeneous group with a high incidence of delirium. To achieve good external validity, we include patients with dementia as well because they are especially prone to develop delirium. Therefore, the results will be widely applicable to the general older hospital population.

The proposed study will contribute to our knowledge because studies on the prophylactic treatment of delirium with follow up remain scarce. Our work may lead to a prophylactic treatment for frail older persons at high risk for delirium that is safe, effective, and easily implementable in daily practice.

## Competing interests

The authors declare that they have no competing interests.

## Authors' contributions

SdeR developed the idea for the study and obtained funding. SdR, BvM, HvO and JK were involved in further developing the idea and designing the trial protocol. AdJ is responsible for the project organisation. AdJ, JG, PK, RvV, RW, CvR contributed to data collection. AdJ, BvM, and SdR were principally responsible for drafting this manuscript. All authors contributed to the final manuscript through critical revision and correction of draft versions, and they approved the final manuscript.

## Pre-publication history

The pre-publication history for this paper can be accessed here:

http://www.biomedcentral.com/1471-2318/11/34/prepub
